# Insulin and Glucagon: Partners for Life

**DOI:** 10.1210/en.2016-1748

**Published:** 2017-01-31

**Authors:** Jens Juul Holst, William Holland, Jesper Gromada, Young Lee, Roger H. Unger, Hai Yan, Kyle W. Sloop, Timothy J. Kieffer, Nicolas Damond, Pedro L. Herrera

**Affiliations:** 1 Novo Nordisk Foundation Center for Basic Metabolic Research and Department of Biomedical Sciences, Faculty of Health Sciences, University of Copenhagen, DK-2200 Copenhagen, Denmark; 2 Touchstone Diabetes Center, Department of Internal Medicine, The University of Texas Southwestern Medical Center, Dallas, Texas 75390; 3 Regeneron Pharmaceuticals, Tarrytown, New York 10591; 4 REMD Biotherapeutics Inc., Camarillo, California 93012; 5 Endocrine Discovery, Lilly Research Laboratories, Indianapolis, Indiana 46285; 6 Department of Cellular & Physiological Sciences, Life Sciences Institute, University of British Columbia, Vancouver, British Columbia V6T 1Z3, Canada; 7 Department of Genetic Medicine & Development, Faculty of Medicine, University of Geneva, CH-1211 Geneva 4, Switzerland

## Abstract

In August 2016, several leaders in glucagon biology gathered for the European Association for the Study of Diabetes Hagedorn Workshop in Oxford, England. A key point of discussion focused on the need for basal insulin to allow for the therapeutic benefit of glucagon blockade in the treatment of diabetes. Among the most enlightening experimental results presented were findings from studies in which glucagon receptor–deficient mice were administered streptozotocin to destroy pancreatic *β* cells or had undergone diphtheria toxin–induced *β* cell ablation. This article summarizes key features of the discussion as a consensus was reached. Agents that antagonize glucagon may be of great benefit for the treatment of diabetes; however, sufficient levels of basal insulin are required for their therapeutic efficacy.

## Teaser

Is glucagon essential for the hyperglycemia in insulin deficiency, as suggested by experimental glucagon antagonism? No, sufficient levels of basal insulin must be present to prevent hyperglycemia.

Hyperglucagonemia and dysregulated glucagon secretion have been implicated in contributing to hyperglycemia in patients with type 1 (1, 2) and type 2 (3–5) diabetes mellitus. These observations have supported continued efforts aimed at understanding the bihormonal relationship between insulin and glucagon and the investigation of glucagon-based therapeutic approaches. Herein, we review much of the seminal work in glucagon biology and highlight recent mechanistic studies that elegantly use the glucagon receptor–deficient mouse model to further assess the ability of blocked glucagon signaling to counteract insulin deficiency.

Glucagon was originally isolated as a hyperglycemic substance (6). When the radioimmunoassays became available (7), it was revealed that glucagon secretion was inversely regulated by plasma glucose concentrations (8), supporting its role as a major glucose-regulating hormone. Studies of fasting patients with type 1 diabetes who were maintained at near euglycemia with insulin infusions demonstrated that plasma glucose and ketone levels increased rapidly after termination of the insulin infusion; importantly, this was paralleled by increases of plasma glucagon concentrations (9). Furthermore, if somatostatin (which strongly inhibits glucagon secretion) was infused simultaneously, the rise in not only glucagon but also plasma glucose and ketones could be strongly reduced, but not when glucagon was replaced (1, 9). These and several other related observations led Unger and Orci (10) to propose in 1975 that diabetic hyperglycemia in general was inseparably associated with inappropriate/unopposed glucagon secretion. This proposal caused considerable debate, particularly regarding the pathophysiology of type 1 diabetes; the existing dogma—that the clinical features of the disease were entirely due to lack of insulin—was not easily abandoned. The strongest support for the traditional belief was from demonstrations of missing or inappropriately low secretion of insulin in patients with diabetic ketoacidosis and the observation of immediate relief of the condition upon insulin administration.

In 1977, Barnes *et al.* (11), using advanced (for the time) assay technology, reported full-scale development of diabetic ketoacidosis in totally pancreatectomized subjects without measurable glucagon secretion, leading the authors to conclude that glucagon is not essential for the development of ketoacidosis in diabetes. At that time, several reports had shown production of glucagon from extrapancreatic sites in experimental animals (in particular cats and dogs) (12), but in Barnes and colleagues’ patients, glucagon levels were immeasurable. This finding suggested that extrapancreatic secretion of glucagon might not occur in people. However, Ravazzola *et al.* (13) identified glucagon-positive cells in the human stomach that showed ultrastructure features consistent with an *α* cell, indicating that humans may indeed have extrapancreatic glucagon, which could contribute to hyperglycemia. Subsequent research with even more sophisticated techniques has now demonstrated that pancreatectomized patients may have remarkably large amounts of glucagon secreted from the gastrointestinal tract (14). Therefore, it remains possible that glucagon can contribute to the diabetic phenotype, even in patients with total pancreatectomy.

For many years, it was tacitly accepted that glucagon might play a role, but that the lack of insulin was believed to be the predominant hyperglycemic factor in diabetes, and this remained textbook dogma. However, the evidence for a role for glucagon was intriguing enough to spur interest in developing antagonists of glucagon action, and many (generally futile) attempts at this were made. Initially, these were mainly peptide-derived glucagon receptor antagonists that were invariably partial agonists, and the antagonistic effect was not sufficiently robust (15). However, evidence started to mount that glucagon might also play a role in the hyperglycemia of type 2 diabetes, including important pioneering studies from the laboratories of Alain Baron (4) and Robert Rizza (16). To provide proof of concept for the use of glucagon antagonism in diabetes, Brand *et al*. (17) normalized blood glucose of mildly alloxan diabetic rabbits (a type 2 diabetes–like model) by administration of a high-affinity monoclonal glucagon antibody, which could effectively neutralize circulating glucagon activity. The same antibody could also normalize glycemia in intermediate-dose streptozotocin (STZ)-diabetic rats (a type 2 model), but it was ineffective in animals with more severe diabetes caused by high-dose STZ (a type 1 model) (18). This supported the general concept regarding the role of glucagon in contributing to hyperglycemia but also indicated that there may be a minimal insulin requirement for glucagon antagonism to be effective.

These and other studies provided further impetus to develop glucagon receptor antagonists, and several pharmaceutical companies became engaged in the hunt. Support for those pursuits was bolstered by findings from studies characterizing a new experimental model: the glucagon receptor (*Gcgr^−/−^*) knockout mouse (19, 20). These animals showed lower blood glucose levels and significantly improved glucose tolerance but displayed similar insulin levels compared with control mice, further supporting the glucagon pathway as a therapeutic target. Soon thereafter, glucagon receptor antisense oligonucleotides (ASOs) were demonstrated to robustly reduce hyperglycemia in several rodent models of type 2 diabetes (21, 22). In agreement with effects of the glucagon-neutralizing antibodies, *Gcgr* null mice or normal animals administered the glucagon receptor ASOs did not develop hypoglycemia; only a mild lowering of fasted and fed plasma glucose concentrations was observed. On the basis of the lack of severe hypoglycemia, the lesson learned was that glucagon antagonism appears relatively safe and therefore a potential therapy for type 2 diabetes. In subsequent years, several small molecule glucagon receptor antagonists were developed and shown to act as powerful (oral) antidiabetic agents in both preclinical and clinical studies of type 2 diabetes (23). The appearance of unexpected side effects [increasing plasma levels of glucagon (see later), hepatic aminotransferases, and low-density lipoprotein (24)] halted development of some but not all of these molecules. Importantly, the clinical results with the antagonists demonstrated unequivocally that inappropriate secretion of glucagon is responsible for a major part of the hyperglycemia of type 2 diabetes (24).

On this background, it was little less than a sensation when in 2011 Lee *et al.* (25) from the laboratory of Roger Unger reported that in *Gcgr^−/−^* mice [developed by Gelling *et al*. (20)], repeat doses of STZ did not cause a diabetic phenotype. The same group subsequently showed (26) that reconstitution of hepatic glucagon receptors in these mice by adenovirus-mediated delivery resulted in a full-blown diabetic state, which resolved as the transgene expression waned. Further studies showed a similar effect in normal mice by using a glucagon receptor antagonist antibody (27). These findings provided new fuel to the old hypothesis that glucagon is responsible for diabetic hyperglycemia, and the old debate flared up again: What is more important—lack of insulin or excess of glucagon?

This discussion was a major theme at a European Association for the Study of Diabetes–sponsored symposium on glucagon recently held in Oxford, England (21st European Association for the Study of Diabetes Hagedorn Oxford Workshop: Glucagon, the Alpha Cell and Intraislet Paracrine Relationships; chairs: Jens Juul Holst and Patrik Rorsman, August 2016), with representatives from many of the groups that had been engaged in this debate and had carried out experimental work to understand these new observations. Several studies were presented, and at the final discussion, a consensus was reached about the probable mechanisms behind the observations from Roger Unger’s and several other laboratories.

First, let us consider more of the important published results on this theme. Thorel *et al.* (28), from the laboratory of Pedro Herrera, produced acute glucagon deficiency by using diphtheria toxin in transgenic animals expressing the human diphtheria toxin receptor under control of the proglucagon promoter. These animals lost almost all of their *α* cells, but the phenotype was unremarkable; however, hyperglycemia after STZ administration was not prevented by the glucagon cell ablation. The authors concluded that the small amount of pancreatic glucagon left after the glucagon cell ablation must be sufficient to maintain the metabolic effects of glucagon, including the type 1–like diabetic phenotype resulting from STZ treatment. Similarly, Steenberg *et al.* (29) also generated acute glucagon cell–depleted mice by using diphtheria toxin, but the massive reduction in pancreatic glucagon cells and glucagon content did not result in improvement of the severe type 1 diabetic phenotype after large doses of STZ. Inspired by the conclusion of Thorel *et al.* (28) that even a small amount of glucagon would suffice to produce the metabolic effects of glucagon, these investigators also administered a glucagon-neutralizing antibody [the same monoclonal antibody as used in the original studies by Brand *et al*. (18)], as well as a glucagon receptor antagonist, previously shown to potently antagonize glucagon action *in vivo* (30). However, neither approach was able to resolve the hyperglycemia.

**Figure 1.**
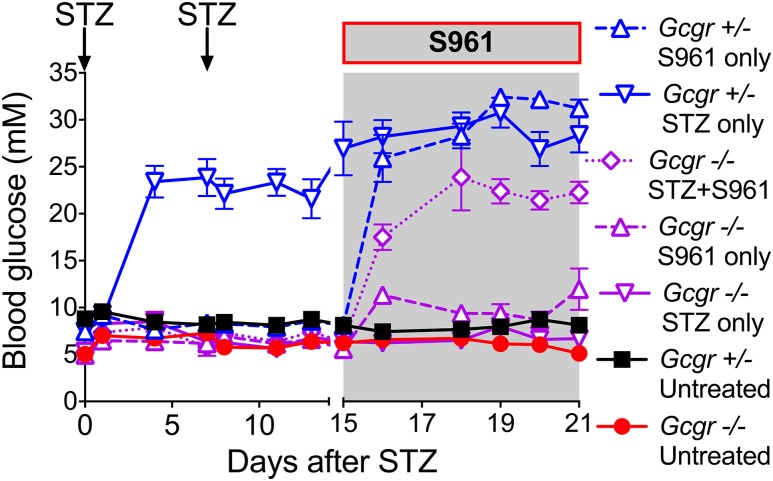
*Gcgr^−/−^* mice become hyperglycemic after efficient insulin signaling blockade. Unlike their *Gcgr^+/−^* counterparts, *Gcgr^−/−^* animals remain normoglycemic after two STZ injections (blue versus purple inverted triangles) but develop hyperglycemia after additional insulin blockade with the insulin receptor antagonist S961 (purple diamonds). Mice were injected with STZ at days 0 and 7 (200 and 150 mg/kg, respectively) to ablate *β* cells and/or treated with S961 between days 15 and 21 (osmotic pump, 40 nmol) to inhibit insulin signaling. Random-fed glycemia is shown. Modified from Damond *et al*. (31).

Additional important contributions to the discussion were data provided by Damond *et al.* in April 2016 (31). These authors hypothesized that incomplete destruction of *β* cells might underlie some of the differences observed in the various prior studies. Using traditional STZ treatment in *Gcgr^−/−^* mice, they reproduced the observations of complete absence of hyperglycemia (Fig. 1, inverted purple triangles). However, realizing that after conventional STZ treatment a residual *β* cell mass was present and thus insulin secretion remains, these investigators used a diphtheria toxin ablation approach that had previously been shown to result in near-total *β* cell ablation (32). Importantly, now after diphtheria toxin–induced ablation, full-blown diabetes developed in the *Gcgr^−/−^* mice. Similarly, insulin blockade with the potent insulin receptor antagonist S961 administered after conventional STZ treatment resulted in development of a full type 1 diabetic phenotype in the *Gcgr^−/−^* mice (Fig. 1, purple diamonds). Similar observations have been made by researchers in the Holland and Unger laboratories using the PANIC ATTAC mouse, a model of triggered *β* cell apoptosis. Here, glucagon receptor antagonist antibodies failed to lower blood glucose in severely diabetic PANIC ATTAC mice (Holland and Unger, unpublished observations). Very recently, Neumann *et al*. (33) assessed mice with double knockout for the insulin and glucagon receptor genes kept alive with exogenous insulin and islet transplants; these studies indicated that the metabolic manifestations associated with complete lack of insulin cannot be overcome by *Gcgr* inactivation and that lack of glucagon signaling was associated with modest reductions in blood glucose and ketones but not survival. Taken together, the cumulative evidence from all of these studies indicates that lack of glucagon signaling efficiently compensates for the consequences of insulin insufficiency, but only if residual insulin action persists after *β* cell loss.

It has been proposed that secretion of *α* cell–generated glucagon-like peptide-1 (GLP-1) might contribute to some of the antidiabetic effects of attenuated glucagon signaling. In agreement with the original findings by Gelling *et al*. (20), Steenberg *et al.* (29) noted that the *Gcgr^−/−^* mice develop massive *α* cell hyperplasia and hypersecretion of glucagon (the same is observed after glucagon-neutralizing antibody, glucagon receptor antagonist antibody, or glucagon receptor ASO administration). Similar findings are made in humans with inactivating mutations in the glucagon receptor (34). Interestingly, the hyperplastic islets in mice also appear to produce GLP-1 (another product processed from proglucagon). This finding is in line with original observations by both the Bloom and Habener laboratories suggesting that proglucagon processing in islets generates some *α* cell–derived GLP-1 (35, 36). The potential involvement of *α* cell–produced GLP-1 in antidiabetic effects was first supported by the glucagon receptor ASO experiments (22) and later by studies with a glucagon receptor antagonist antibody (37). The role of GLP-1 in glucose lowering that results from blunting glucagon signaling was investigated by Jun *et al.* (38) from the Eli Lilly and Co. laboratories using a double-knockout mouse model harboring deletion of both *Gcgr* and *Glp1r*. Similar to Unger’s findings, these studies showed that STZ-induced diabetes did not develop in *Gcgr^−/−^* mice. However, in the double-knockout mice, the same STZ treatment resulted in substantial hyperglycemia, amounting to about half of that seen in control mice given STZ (38). Also, administration of a glucagon receptor antagonizing antibody reversed hyperglycemia in STZ-treated mice [in agreement with results from Wang *et al.* (27) using a different antibody], but importantly, the antibody was only partially effective in *Glp1r^−/−^* mice (38). Judging from these results, actions exerted *via* the GLP-1 receptor appear to play an important role in the glucose-lowering phenomenon. Therefore, increased *α* cell–produced GLP-1 might be responsible for some of the antidiabetic effects of glucagon blockade, independent of its insulinotropic actions. Traditionally, GLP-1 is not thought to influence hepatic glucose production, but this has been challenged in recent studies (39), in which GLP-1 appeared to directly inhibit hepatic glucose production in mice and humans (40, 41).

Can the GLP-1 hypothesis also explain part of the efficacy demonstrated by glucagon receptor antagonist antibodies, which appear to be nearly as effective as *Gcgr* knockout in preventing STZ-induced hyperglycemia? As mentioned earlier, there is little doubt that strong glucagon antagonism results in *α* cell hyperplasia and hyperglucagonemia in rodents (22, 38) and nonhuman primates (42). Prevention of glucagon action by glucagon receptor antibodies in humans would, therefore, be expected to have similar results. With the currently available data, it is unclear whether there are significant differences in the degree and rate of *α* cell proliferation induced by glucagon receptor antagonist antibodies in rodents versus primates, and the implications of *α* cell hyperplasia following long-term treatment need to be carefully assessed. Furthermore, the kinetics of GLP-1 secretion by *α* cells after blocking of glucagon signaling also needs investigating.

**Figure 2.**
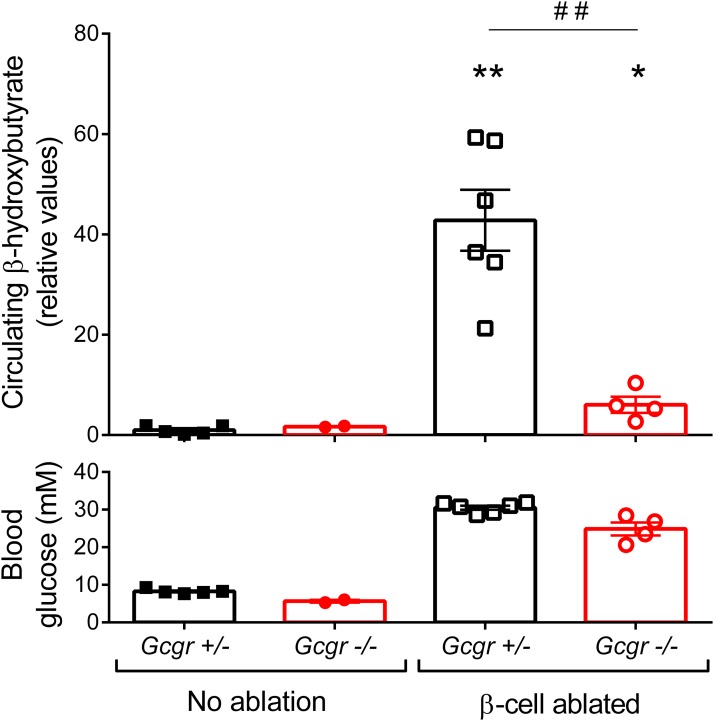
The lack of glucagon action mitigates hyperketonemia development after *β* cell ablation. In mice with normal glucagon signaling, diphtheria toxin–mediated *β* cell destruction leads to a sharp increase in circulating levels of ketone bodies. This increase is attenuated but not entirely abolished in *β* cell–ablated *Gcgr^−/−^* mice. The corresponding random blood glucose levels averaged over 1 month are shown on the lower panel. Experimental procedures were performed as described (31). Briefly, adult (10–12 weeks old) *RIP-DTR;Gcgr^−/−^* males (20, 31) were injected with diphtheria toxin to induce *β* cell ablation. One month later, *β*-hydroxybutyrate levels were measured from plasma using an enzymatic assay (MAK041, Sigma-Aldrich). Error bars indicate SEM. *P < 0.05 and **P < 0.01 versus unablated *Gcgr^+/−^* mice; ## *P* < 0.01; *β* cell–ablated: *Gcgr^+/−^* versus *Gcgr^−/−^*, Mann-Whitney *U* test.

The consensus conclusion from the total body of work is that complete lack of insulin, as in severe long-standing type 1 diabetes, locks the liver in a state where blunting of glucagon action is unable to downregulate glucose production. However, this applies only to conditions of severe insulin deficiency. Experimentally, a threshold seems to exist in the severity of diabetes, above which only insulin or combinatorial treatment can effectively normalize blood glucose. Perhaps this threshold is coincident with ketosis, as *β* cell depletion sufficient to induce ketosis cannot be completely restored by glucagon antagonism. The control of ketogenesis is equally importantly regulated, and some observations suggest that ketogenesis may be more selectively dependent on glucagon action than glucose production (43). It is important to note that ketosis was reduced in further studies of the diphtheria toxin *β* cell–ablated *Gcgr^−/−^* animals [Damond *et al.* (31); Fig. 2] in agreement with observations that *Gcgr* gene deletion attenuated hyperketonemia in *InsKO* pups (33). Given the important role of glucagon signaling in regulating hepatic lipid oxidation (44), it will be important to determine whether chronic treatment with glucagon antagonists promotes hepatosteatosis.

In the entire interval of relative insulin insufficiency, which covers late-stage type 2 diabetes and probably also type 1 diabetes with residual insulin production, (inappropriate) glucagon levels are responsible for hepatic glucose production to an extent, where a complete normalization of glucose levels may be achieved if glucagon actions are prevented. Therefore, glucagon antagonism remains an important target for antidiabetic therapy in conditions where basal insulin action remains and should be further investigated (44).

## Abbreviations:

ASO: antisense oligonucleotides
GLP-1: glucagon-like peptide-1
STZ: streptozotocin.
